# Brachial plexus block using lidocaine/epinephrine or lidocaine/xylazine in fat-tailed sheep

**Published:** 2013

**Authors:** Safoura Ghadirian, Nasser Vesal

**Affiliations:** Department of Veterinary Clinical Sciences, School of Veterinary Medicine, Shiraz University, Shiraz, Iran.

**Keywords:** Brachial plexus, Epinephrine, Lidocaine, Sheep, Xylazine

## Abstract

This blinded, randomized experimental study was designed to evaluate the analgesic effects of adding epinephrine or xylazine to lidocaine solution for brachial plexus block (BPB) in sheep. Nine healthy, fat-tailed female lambs (26.6 ± 1.5 kg) were randomly allocated into three groups: lidocaine 2%, 5 mg kg^-1^ (LID, n = 6), lidocaine (5 mg kg^-1^) with epinephrine 5 µg mL^-1^ (LIDEP, n = 6) or lidocaine (5 mg kg^-1^) with xylazine 0.05 mg kg^-1^ (LIDXY, n = 6). Each animal was tested twice. The sheep received a total volume of 0.25 mL kg^-1^ for BPB. A nerve stimulator was used to locate the nerves of the brachial plexus. Onset and duration of analgesia of the forelimb were evaluated using superficial and deep pin prick and pinching of skin with a hemostat clamp. Heart and respiratory rates, and rectal temperature were recorded before and at predetermined intervals following the completion of the block. Brachial administration of LID, LIDEP or LIDXY produced forelimb analgesia within 11.3, 11.0 and 7.0 minutes, respectively. The mean duration of analgesia was 100.0 min in LID and 133.2 min in LIDEP group. The mean duration of analgesia in LIDXY group (186.8 min) was significantly longer compared with LID group. In LIDEP group a significant increase in heart rate occurred 5 min after drug administration. Heart rate decreased from 35 to 80 min in sheep received LIDXY. In conclusion**, **the addition of xylazine to lidocaine solution for BBP provided a prolonged duration of action without any adverse effects in fat-tailed sheep.

## Introduction

In ruminants, surgical procedures are commonly performed under sedation and judicious use of local or regional anesthetic techniques. Local and regional anesthetic techniques are preferred over general anesthesia in food animals because they produce minimal cardiopulmonary alterations, require limited amount of equipment, minimize veterinary supervision, and lower the cost of the procedure.^1^ Brachial plexus block (BPB), as a regional analgesic technique, is sometimes used in veterinary patients to provide analgesia for animals undergoing surgery of the distal forelimb. This technique can play an important role in the perioperative period and as a part of a balanced anesthetic protocol.^2^

Although BPB was first described in 1951 by Tufvesson as a means of providing anesthesia of the forelimb in dogs, there are only few reports of its clinical^3^^-^^5^ or experimental^6^^-^^10^ use in animals. In human medicine, a BPB, alone or in combination with general anesthesia, is commonly used to provide surgical anesthesia and postoperative analgesia of the upper limb.^11^^,^^12^

Brachial plexus block can be achieved by injecting local anesthetic around the brachial plexus nerves at the level of the scapulohumeral joint.^13^ Peripheral nerve stimulators have been employed to accurately locate the radial, median, ulnar, musculocutaneous, and axillary nerves, thereby reducing the dose of local anesthetic and enhancing the chances of successful BPB in small animals.^3^^-^^5^^,^^9^^,^^10^^,^^14^

Lidocaine, an intermediate-acting agent, is the most frequently used local anesthetic solution for regional anesthesia in veterinary medicine, which causes a blockage of the sensory and motor fibers for a duration of 1.0-1.5 hr.^15^ Regional anesthesia of the brachial plexus with longer-acting drugs or drug combinations may provide an excellent means to control postoperative pain after fore-limb surgery in ruminants. 

Two major groups of drugs used to increase the depth or duration of local anesthetics for epidural block are vaso-constrictors (mainly epinephrine) and α_2_-adrenergic agonists. Xylazine is an α_2_-adrenoceptor agonist that is used as a sedative in veterinary practice. Xylazine alone or in combination with lidocaine has been used for epidural or subarachnoid anesthesia in a variety of species and has been demonstrated that their combination induces more prolonged analgesia than that observed with the use of either drug alone. It appears to exhibit direct local anesthetic sensory and motor nerve blocking actions in addition to its spinal cord α_2_-adrenoceptor-mediated analgesic effects.^1^

The effect of adding α_2_-agonists, clonidine and dex-medetomidine, to local anesthetics for BPB has been studied in man and the results demonstrated that α_2_-agonists improve quality and duration of a local anesthetic block.^11^^,^^16^^-^^19^

To the best of the authors’ knowledge, the effect of adding xylazine to lidocaine for BPB has not been reported previously. The objective of the study reported here was to evaluate analgesic efficacy of BPB induced with lidocaine/ epinephrine or lidocaine/xylazine combinations in fat-tailed sheep. Plain lidocaine has been included for comparison.

## Materials and Methods

The Institutional Animal Care and Use Committee approved the protocol for this project. Nine healthy, 5 to 6 month-old (5.6 ± 0.3 month; range 5.2 - 6.0), fat-tailed Ghezel female lambs, with a body condition score of 2.8 ± 0.3 (Range 2.5 - 3.0; on a scale of 0 - 5 units) and with a mean (± SD) weight of 26.6 ± 1.5 kg range, 24 - 29 kg were included in the present study. 

The sheep were confined to indoor pens and were given alfalfa, concentrate (grain mix) and water *ad libitum* and were allowed an acclimation period of two weeks prior to the beginning of the study. Health status was established on the basis of a thorough physical examination and normal complete blood count and total protein. Fecal sample examination revealed no parasite infestation. Sheep were randomly assigned to one of three groups (six sheep per group) and received lidocaine HCl 2% (Caspian Tamin Pharmaceutical Co., Rasht, Iran) (LID), lidocaine 2% with 5 µg mL^-1^ epinephrine (Darou Pakhsh Co., Tehran, Iran) (LIDEP) or 2% lidocaine with 0.05 mg kg^-1^ xylazine HCl 2% (Alfasan, Woerden, The Netherlands) (LIDXY). Lidocaine- epinephrine and lidocaine-xylazine solutions were freshly prepared by adding epinephrine (1:1000) or xylazine to lidocaine immediately before use. The total volume of drug(s) injected was 0.25 mL kg^-1^. The pH of the anesthetic solutions was determined using a laboratory pH meter (Crison pH meter; Basic 20^+^, Barcelona, Spain). 

The right limb was selected for block and each of the nine sheep was used twice, with at least a 7-day interval between experiments. Animals were fasted for 12 hr before experiment, but water *ad labium*. 

On the morning of the trials, baseline values for heart rate, respiratory rate, and rectal temperature were recorded and normal motor function of the thoracic limb during walking was confirmed. Subsequently, sheep were restrained in left lateral recumbency and the skin area medial to the right scapulohumeral joint was clipped and aseptically prepared. A nerve stimulator (Stimuplex; Braun, Melsungen, Germany) was used to locate the nerves of the brachial plexus. The negative electrode from the nerve stimulator was attached to the proximal portion of a 22-gauge, 7.5-cm insulated needle (Pajunk, Geisingen, Germany); the positive electrode was attached to the skin at a distance of 5-7 cm from the shoulder joint. The insulated needle was inserted through a small stab puncture at a point medial to scapula-humeral joint, advancing toward the costochondral junction of the first palpable rib, medial to the scapula but outside the thorax. Following needle insertion, the nerve stimulator was turned on and the current of the nerve stimulator was initially set at 5 mA. As muscle contractions of the limb and extension/flexion twitches were observed, the current was gradually decreased and the needle was manipulated to obtain a maximal twitch with as little current as possible (0.2-0.3 mA, 0.1 msec and 2 Hz). After aspiration for blood or air to confirm that the needle was not placed in a blood vessel or in the thorax, a small amount of anesthetic solution (0.5-1.0 mL) was injected slowly until the twitch disappeared. In order to block all branches of the brachial plexus, this technique was performed several times, fanning the needle dorsal and ventral from the initial placement. The remainder of anesthetic solution was injected while withdrawing the needle. The time required to complete the procedure was recorded. Following administration of the anesthetic solution, sheep was placed in a canvas sling in a standing position.

Time to onset and duration of complete analgesia (sensory block) of forelimb within and below the elbow joint were recorded based on the complete absence of response to painful stimuli including superficial and deep pin prick with a 25-gauge needle and pinching of skin with a hemostat clamp closed to the first ratchet. In order to determine the time to onset, analgesic testing was performed every 30 sec after the completion of the injection. Analgesia was evaluated every 10 min until a response was observed. Onset and duration of right forelimb paralysis (motor block) were also determined by manually flexing the hind limbs to evaluate muscle tone and the animal's ability to support its own weight. The same investigator assessed analgesia in all cases, and was blinded to the treatment given. 

Heart rate (HR) was measured by counting the heart beats over the cardiac area using a stethoscope. Respiratory rate (RR) was measured by counting chest movements per min and rectal temperature (RT) was measured with a digital thermometer. Heart rate, respiratory rate, and rectal temperature were measured before drug administration (time 0) and 5, 10, 15, 20, 30, 40, 50, 60, 75 min after drug injection and every 15 min until the end of analgesia.

**Statistical analysis. **The data were evaluated for normal distribution using the Kolmogorov-Smirnov test. HR, RR, and rectal temperature values were compared using analysis of variance (ANOVA) for repeated-measures with time and group as factors, followed by Duncan’s test. A one-way ANOVA followed by Duncan’s test was used to compare onset and duration of sensory and motor (paralysis) blocks and the time required to perform the procedure. Statistical analysis was undertaken using a SPSS (Version 10 for Windows, MicroMaster, Richboro, PA, USA) and *p *≤ 0.05 was considered significant. All data are presented as mean ± SD for each treatment group.

## Results

There were no significant differences in body weight and the mean time for drug injection between groups (Table 1).

No difficulty was encountered in locating the proper site for injection of local anesthetic and the procedure was completed within 5 min. 

**Table 1:** Descriptive data, injection time and drug's pH in lidocaine (LID), lidocaine-epinephrine (LIDEP) and lidocaine-xylazine (LIDXY) groups

**Parameters**	**Treatments**		
	**LID**	**LIDEP**	**LIDXY**
**Number of animals**	6	6	6
**Body weight (kg)**	25.0 3.0	26.0 2.0	27.0 2.0
**Time (min)**	3.9 0.5	4.0 0.6	4.3 0.3
**pH**	6.28	6.27	6.26

Time = Time required to perform the procedure.

**Fig. 1:**
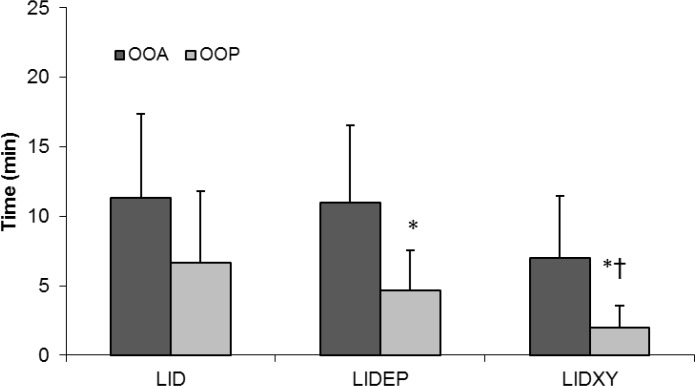
Time to onset of limb analgesia (OOA) and paralysis (OOP), (Mean   SD) after brachial plexus blockade using lidocaine (LID), lidocaine-epinephrine (LIDEP) or lidocaine-xylazine (LIDXY) in fat-tailed sheep (n = 6).

**Fig. 2:**
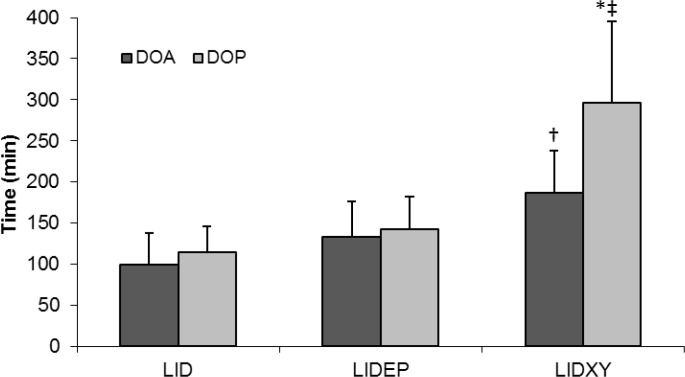
Duration of limb analgesia (DOA) and paralysis (DOP), (Mean   SD) after brachial plexus blockade using lidocaine (LID), lidocaine-epinephrine (LIDEP) or lidocaine-xylazine (LIDXY) in fat-tailed sheep (n = 6).

There were no significant differences in onset of analgesia among groups but onset of paralysis was significantly faster in LIDXY group compared to that of LID group. Significant difference was not detected between time to onset of analgesia and paralysis in LID group (11.3 ± 6.1 vs. 6.7 ± 5.1 min) (*p *> 0.05), but onset of limb paralysis was significantly faster than onset of analgesia in LIDEP and LIDXY groups (*p *< 0.05), (Fig. 1). Duration of analgesia was significantly longer (*p *< 0.05) in LIDXY group compared to that of LID group (Fig. 2). Lidocaine-xylazine combination produced significantly longer limb paralysis compared to those of other groups. Two sheep (one in lidocaine and one in LIDEP group) failed to achieve complete block within 45 min after the injection. Complete BPB was subsequently achieved in these animals one week later.

**Fig. 3:**
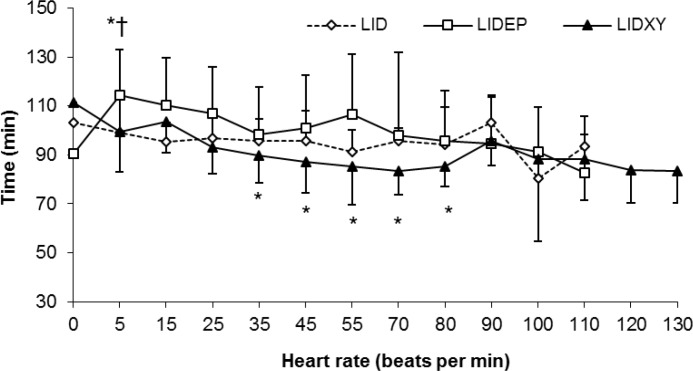
Heart rate (Mean   SD) after brachial plexus blockade using lidocaine (LID), lidocaine-epinephrine (LIDEP) or lidocaine-xylazine (LIDXY) in fat-tailed sheep (n = 6).

In LIDEP group a significant increase in HR occurred 5 min after drug administration when compared with base-line and other groups (*p *< 0.05), (Fig. 3). No change in HR was observed in LID group (*p *> 0.05). However, HR was decreased significantly from 35 to 80 min in sheep received LIDXY. No change in RR and RT (data not shown) were observed in all three groups. Mild sedation and frequent urination were observed in sheep received LIDXY. 

No adverse reactions associated with administration of drugs or obvious signs of local anesthetic toxicity (extensor rigidity, muscle twitching and convulsions) were encountered after performance of BPB in any of the sheep in this study.

* Significant differences from OOA (*p *< 0.05).

† Significant differences from LID group (*p *< 0.05).

* Significant differences from DOA (*p *< 0.05).

† Significant differences from LID group (*p *< 0.05).

‡ Significant differences from LID and LIDEP groups (*p *< 0.05).

* Significant differences from baseline (*p* < 0.05).

† Significant differences from LID and LIDXY groups (*p* < 0.05).

## Discussion

Local anesthetics have the unique ability to block completely the sensation of pain and have been used clinically as adjuncts to light general anesthesia in both small and large animals.^15^ Regional anesthesia decreases general anesthetic requirement, improve recovery and prevent central sensitization of nociceptive pathway after painful surgical procedures, reducing the requirements for postoperative analgesia.^20^^,^^21^ Local and regional anesthetic techniques may be used before, during, or after surgery to provide postoperative analgesia. Blockade of the brachial plexus has been used extensively for surgery of the forearm and hand in humans.^11^^,^^12^ This technique provides excellent intraoperative anesthesia as well as postoperative analgesia and may reduce postoperative analgesic requirement.^21^ Brachial plexus block is also a classic technique used for procedures that are performed on the forelimbs in animals.^22^ Brachial plexus block in sheep can be achieved by desensitizing the ventral roots of the 6^th^, 7^th^, and 8^th^ cervical (C_6_, C_7_, and C_8_) and 1^st^ thoracic (T_1_) spinal nerves as they enter the axillary space.^23^

Various techniques including blind needle placement, nerve stimulator-guided or ultrasound-guided have been reported for performing a BPB.^24^^-^^25^ Peripheral nerve stimulators have been used to accurately locate the branches of brachial plexus, thereby reducing the required dose of local anesthetic and enhancing the success rate of the BPB. The overall success rate of BPB in the present study was about 89.0%. In goats, success rate of 20.0% and 95.0% has been reported using blind and nerve stimulation techniques, respectively.^25^ The total volume of local anesthetic solution injected plays an important role in the effectiveness and success of BPB.^26^^-^^27^ Total volumes of 0.25-1.0 mL kg^-1^ in dogs^3^^,^^7^^,^^9^^,^^10^^,^^28^ and 0.2-0.6 mL kg^-1^ in cats^5^^,^^22^^,^^29^ were used. It has been suggested that a volume of 0.3 mL kg^-1^ might be adequate when performing BPB in dogs.^26^ Total volume of 40 mL of 2% lidocaine per adult cattle,^1^ 10 mL of 2% lidocaine for calves weighing 62 - 94 kg,^30^ 0.3-0.4 mL kg^-1^ for goat^25^ and 0.44 mL kg^-1^ for sheep^8^ have been reported. The total volume of local anesthetic solution required for successful BPB has not been determined in sheep. In the present study, the total volume was 0.25 mL kg^-1^ for lidocaine alone and its combination with epinephrine or xylazine. A higher volume of local anesthetic may increase the likelihood of successful BPB. 

The results of the study presented here showed that xylazine prolonged both motor and sensory block of the BPB induced by lidocaine. Several studies in human, indicated that α_2_-adrenergic agonists (clonidine and dexmedetomidine) increase the duration of analgesia when added to local anesthetics for brachial plexus nerve block.^11^^,^^16^^-^^19^^,^^31^^,^^32^ Although α_2_-adrenergic agonists have been used to prolong spinal and epidural anesthesia in a variety of animal species and to the best knowledge of the authors, there is no report on the use of α_2_-adrenergic agonists for BPB in animals.

The exact mechanism of the prolongation of local anesthetic activity by α_2_-adrenergic agonists when injected at peripheral nerve sites is unknown. However, three mechanisms have been suggested. First, local vasoconstriction induced by α_2_-adrenergic agonists may interfere with vascular absorption of local anesthetics, resulting in higher concentrations at the nerve and lower plasma levels of local anesthetic. However, several clinical studies demonstrated that clonidine, in contrast to epinephrine, does not reduce plasma local anesthetic concentrations following brachial or cervical plexus blocks.^16^^,^^33^^-^^34^

Second, α_2_ adrenergic agonists may induce analgesia via a systemic mechanism following vascular absorption. However, systemic administration of clonidine had no effects on the duration of local anesthetic block,^35^ suggesting that prolongation of BPB by α_2_-adrenergic agonists is independent of systemic effects. Clonidine, as the sole analgesic, provided better and longer postoperative analgesia after administration to the interscalene plexus compared with subcutaneous administration of the same dose in patients undergoing shoulder arthroscopy, indicating a direct peripheral analgesic action of clonidine.^34^

Third, α_2_-adrenergic agonists may have a direct local action on peripheral nerves. Although intrathecally and epidurally administered α_2_-adrenergic agonists are believed to produce analgesia through activation of α_2_-adrenergic receptors in the spinal cord,^1^ the exact mechanism by which α_2_-adrenergic agonist potentiates local anesthetic peripheral nerve block has not been precisely elucidated. Experimental studies have shown that clonidine blocks conduction of C and A δ fibers and intensifies conduction block of local anesthetics in isolated neurons.^36^^,^^37^

A more recent study, using sciatic nerve block in rats, demonstrated that prolongation of duration of local anesthetic nerve blockade by clonidine is not mediated by an α_2_-adrenergic mechanism but likely involves blockade of hyperpolarization-activated cation current in peripheral nerve fibers.^38^ However, clonidine and dexmedetomidine, a more selective α_2_-adrenoceptor agonist, have been reported to enhance the local anesthetic action of lidocaine via peripheral α-_2A_ adrenoceptor subtypes when injected intracutaneously in guinea pigs.^39^

In the present study, the duration of anesthesia of the forelimb tended to be longer with lidocaine-epinephrine when compared with lidocaine alone (133.2± 43.3 vs. 100.0 ± 39.9 min), but no statistical significance was achieved. We had expected that lidocaine containing epinephrine would result in prolonged analgesia because of local vaso-constrictive effect of epinephrine. The lack of a significant difference between LID and LIDEP groups in the present study might have been attributed to the relatively small sample size of each group or a low concentration of epi-nephrine used with lidocaine in this study. Epinephrine is effective when added to anesthetic solution in concentration of 5-20 µg mL^-1^.^15^ However, the optimal concentration of epinephrine for BPB is not known. The lowest recommended concentration of epinephrine (5 µg mL^-1^) was used in the present study. It has been reported that the addition of clonidine to bupivacaine for brachial plexus nerve block results in a significantly longer duration of analgesia as compared to the addition of epinephrine.^40^ However, in another study, onset and recovery of sensory and motor block were not different following addition of either clonidine or epinephrine to lidocaine for BPB.^33^

In this study, onset of limb paralysis was significantly faster than onset of analgesia in LIDEP and LIDXY groups. The duration of limb paralysis was also significantly longer than duration of analgesia in LIDXY group. It has been demonstrated that BPB following the administration of lidocaine or bupivacaine produce more rapid onset of motor block when compared with sensory block in dogs and goats.^9^^,^^25^ Shorter onset time and longer duration of motor block were observed with bupivacaine and ropivacaine in dogs.^14^ This phenomenon has been explained by the somato-topical arrangement of nerve fibers in mixed peripheral nerves. When a mixed peripheral nerve is exposed to local anesthetic solution, the diffusion of the drug occurs from the outer surface (mantle) toward the center (core) of the nerve along a concentration gradient. Therefore, motor nerves located in the outer mantle of the mixed nerve are blocked first. Consequently, skeletal muscle paralysis may precede the onset of sensory block. Since the vascular supply is usually centrally located near the core of the mixed nerve, removal of local anesthetics during recovery occurs primarily in the core and the sensory block is usually shorter than motor block.^15^

Transient increase in HR was observed in sheep given LIDEP, suggesting rapid systemic absorption of epinephrine from the injection site following drug administration. In a previous study, a significant increase in HR and arterial blood pressure have been reported within the first 6 min after brachial plexus nerve block using lidocaine containing 5 µg mL^-1^ epinephrine.^43^ In fact, the vaso-dilatory effect of lidocaine may accelerate the systemic absorption of epinephrine.^44^

Sedation, bradycardia and increased urination observed in LIDXY group are common side effects following xylazine administration in ruminants, indicating systemic drug absorption from the injection site. Sedation occurred in human patients receiving clonidine for BPB.^16^^,^^17^^,^^31^^,^^32^ Xylazine-induced sedation may be beneficial during surgical procedures in non-anesthetized animals. The decrease in HR is attributed to increased parasympathetic activity and decreased sympathetic outflow from CNS.^45^ The mechanism of increased urination frequency after LIDXY administration may be associated with inhibition of antidiuretic hormone release from the pituitary and the osmotic diuretic effect of hyperglycemia induced by xylazine.^45^

In conclusion, xylazine (0.05 mg kg^-1^) added to lidocaine for BPB prolonged motor and sensory block durations without producing any clinically important adverse reactions. This may contribute to pain relief in the immediate postoperative period in animals undergoing forelimb surgery. Further studies are required to determine the optimum doses of xylazine as an adjuvant to lidocaine for BPB in sheep.
